# Implementing the Synergy Model: A Qualitative Descriptive Study

**DOI:** 10.3390/nursrep12010011

**Published:** 2022-02-14

**Authors:** Charissa P. Cordon, Pamela Baxter, Ari Collerman, Kirsten Krull, Celia Aiello, Jennifer Lounsbury, Maura MacPhee, Sonia Udod, Kim Alvarado, Tim Dietrich, Noori Akhtar-Danesh, Meena Ramachandran, Natalie Meisenburg

**Affiliations:** 1 Hamilton Health Sciences, Hamilton, ON L8L 2X2, Canada; 2 School of Nursing, McMaster University, Hamilton, ON L8S 4L8, Canada; 3 School of Nursing, University of British Columbia, Vancouver, BC V6T 2B5, Canada; 4 College of Nursing, University of Manitoba, Winnipeg, MB R3T 2N2, Canada; 5 School of Physical and Occupational Therapy, McGill University, Montreal, QC H3A 0G4, Canada

**Keywords:** nursing model of care, care delivery redesign, Synergy Model, patient needs assessment, facilitators and barriers

## Abstract

Hospitals across our nation are seeking to implement models of care that meet the primary goals of Quadruple Aim: Improved population health, cost-effective care delivery, and patient and provider satisfaction. In an effort to address the Quadruple Aim and our patients’ care needs, Hamilton Health Sciences (HHS) embarked on a model of care delivery redesign, beginning with nursing care delivery. From 2013 to 2018, 12 clinical programs at HHS implemented the Synergy Model with its accompanying synergy patient needs assessment tool for nurses to objectively assess patients’ acuity and dependency needs. Data on patients’ priority care needs were used to inform a nursing model of care redesign at HHS, including skill mix and staffing levels. This five-year project was an organization-wide quality improvement initiative. As part of the evaluation, HHS leaders partnered with health services nurse researchers to conduct a mixed methods study. This paper describes the evaluation outcomes from the qualitative component of the study, which included interviews with clinical nurse leaders and direct care nurses. Data were analyzed using descriptive thematic analysis. Some key findings were increased nurse awareness of patients’ holistic care needs and leaders’ capacity to plan staffing assignments based on patients’ priority care needs. Themes helped inform recommendations for key stakeholders, including nurse leaders and direct care nurses.

## 1. Introduction

Hospitals across our nation are seeking to implement models of care that meet the primary goals of Quadruple Aim: Improved population health, cost-effective care delivery, and patient and provider satisfaction [1]. A model of care is broadly defined as the way health care services are delivered [2]. An effective model of care selection is based on the patient population, staff skill mix and availability, economic feasibility, and intended patient outcomes. The model of care must ensure that patients receive the right care, at the right time, by the right team, and in the right place [3]. The need for effective models of care is more important than ever with the increasingly complex patient care needs of our ageing population, and nurses being asked to do more with less.

In an effort to address the Quadruple Aim and our patients’ care needs, Hamilton Health Sciences (HHS) embarked on a model of care redesign, beginning with nursing care delivery. The Synergy Model was selected as the nursing model of care [4]. This model was originally developed for use in critical care settings in the United States, but it has since been used in a variety of healthcare sectors in the United States and other countries [5,6,7]. In Canada, the Synergy Model has been successfully used in acute care and inpatient specialty areas [8,9] and in emergency services [10,11]. The premise of this model is that synergy or best possible outcomes arise from a ‘fit’ between patients’ needs and nursing competencies [4]. There is a growing body of evidence that the Synergy Model can be effectively applied across different healthcare settings while demonstrating significant positive outcomes that align with the Quadruple Aim goals for patients and nurses [5].

The Synergy Model includes the synergy patient assessment tool, which has a 5-point Likert-like scale ranging from scores of 1–2 (low needs) to 3 (moderate needs) and 4–5 (high needs). The scoring system can be reversed so that scores of 1–2 are high needs and scores of 4–5 are low needs. The synergy patient assessment tool is accompanied by scoring guidelines that consist of measurable assessment indicators that nurses use to differentiate the level of care needs for their patients. The assessment indicators are objective indicators that nurses routinely use to assess their specific patient populations, such as neurology, surgery, and cardiology. After completing their patient assessments, nurses use the synergy patient assessment tool with its scoring guidelines to score each of their patients as low, moderate, or high acuity and as low, moderate, or high capability. Acuity characteristics include complexity, stability, predictability, resilience, and vulnerability. These characteristics reflect the need for different levels of nursing intervention. The capability characteristics are the capacity to do own activities of daily living, capacity to make cogent decisions, and access to resources for discharge and care at home. Capability needs do not always require nursing care interventions [4].

Synergy scores yield objective patient assessment information that can be used to make more effective and accurate staffing assignments in real time. Scores can also be averaged and graphed to look for trends in patient population care needs over different lengths of time, such as over a shift, a week, a month, or seasonally. Because the Synergy Model is nurse driven, use of this model has been associated with nurse empowerment and more effective relational practice [9,10,11,12,13,14] while the patient-centered approach to patient needs assessment has been linked to more effective patient outcomes [5].

### Background of Synergy Model Implementation at Hamilton Health Services

From 2013 to 2018, 12 clinical programs at HHS implemented the Synergy Model as a quality improvement initiative to enhance patient care delivery by optimizing the use of available direct care nurses. To implement the Synergy Model across a diverse range of settings in HHS, we used a generic care delivery design toolkit based on the work by MacPhee and her colleagues [13]. Even though clinical programs and patient populations vary, the eight patient needs characteristics of the Synergy Model are ‘universals’, and regardless of the site or population, the five-point scoring system conveys the same level of priority care needs that nurses and leaders can readily use to inform their care delivery decisions. The use of a generic toolkit has also facilitated the nursing model of care redesign across diverse healthcare sectors in numerous international settings [5,9].

Given the significant organizational and staff investment in a new nursing model of care, an HHS senior-level decision was made to garner feedback on the initial implementation from nurse leadership and direct care nurses. A collaboration was forged between HHS nurse leadership and nurse researchers to conduct a mixed methods study on Synergy Model implementation to better inform ongoing implementation strategies within the organization. This study is the qualitative descriptive component of the larger study. We believe these study findings will be useful to other healthcare organizations and their leadership who are considering a nursing model of care redesign.

Our research questions are: (1) What are the reasons for implementing the Synergy Model? (2) What are the barriers and facilitators during the implementation of the Synergy Model? and (3) What are the participants’ perceived outcomes after implementation of the Synergy Model?

## 2. Materials and Methods

The qualitative component of a larger mixed methods study employed a qualitative descriptive study design [15]. Qualitative descriptive research methods are used to obtain basic information about informants’ perspectives of a particular intervention or phenomenon [16]. This design was chosen to better understand nurses’ and nurse leaders’ experiences with the Synergy Model. Ethics approval was obtained from the HHS Research Ethics Board #7998.

The setting was one tertiary care institution in a large urban area in eastern Canada. Within HHS, 12 clinical areas implemented the Synergy Model from 2013 to 2018. The 12 clinical areas were: hematology, oncology, surgical oncology, alternate levels of care, neurosurgery, cardiac surgery, cardiovascular surgery, acquired brain injury, post anesthesia care unit, the general medical unit, and the medically complex care unit.

Criterion sampling [17] was used to seek out nurse leaders and direct care nurses within the 12 clinical programs using the Synergy Model. For the purposes of this research, ‘unit leader’ refers to managers, clinical leaders, charge nurses, advanced practice nurses, and educators; and ‘direct care nurse’ refers to registered nurses (RNs) and registered practical nurses (RPNs). Our study aim was not to explore differences in perspectives between nurses and unit leaders. The purpose of our sampling strategy, therefore, was to obtain representative rich descriptions across the 12 clinical programs of the factors that influenced adoption, implementation, and outcomes related to the Synergy Model [16,17]. Our sampling goal was to interview nurses and nurse leaders most familiar with the implementation process and outcomes. Posters describing the research were placed in central locations on each unit and a study cover letter with a consent form was emailed to unit leaders and direct care nurses in the 12 clinical programs. Potential participants were asked to contact the research assistant by email, who provided additional information and obtained participants’ informed consent.

A draft interview guide was developed for the research questions and piloted with three volunteer RNs who were not part of the study. The research assistant conducted semi-structured interviews of 45–60 min each with 19 participants (12 unit leaders and 7 direct care nurses) via telephone. All interviews were digitally audio-recorded and transcribed using Sonix ^®^ (Sonix ^®^, San Francisco, CA, USA) software. Per ethics, volunteer participants were de-identified and their transcripts were labeled and numbered as ‘direct care nurse’ (1, 2, etc.) or ‘unit leader’ (1, 2, etc.). Due to the small sample size in one institution, demographics were not collected.

Descriptive thematic analysis [18] was used by two research team members who independently read and deductively/inductively content analyzed the transcripts for descriptive themes pertaining to each of the research questions. To establish study rigor [17], descriptive thematic codes were independently established by the researchers and discussed for consensus. A codebook was developed to promote inter-rater reliability. New descriptive themes were added and existing ones modified through regular discussions between the researchers. The coding framework, coded transcripts, and researcher notes were systematically organized within NVivo 12 (QSR International, Burlington, NJ, USA) software for transparency and for the researchers’ review. Across the 19 participants, there was representation for each clinical program of either a direct care nurse or unit leader. Saturation of themes was reached across these 19 participants.

## 3. Results

Table 1 is a summary table of the themes by the research question. The following sections and sub-sections present the descriptive themes per research question with exemplar quotes by unit leaders and direct care nurses.

### 3.1. Reasons for Adopting the Synergy Model

The two key themes for the first question were: (1) Exploring staffing models, and (2) supporting equitable workloads.

#### 3.1.1. Exploring Staffing Models

Direct care nurses and leaders indicated that one of the reasons for adopting the Synergy Model was to better address nursing clinical program issues, such as increased nursing turnover and increased patient acuity.

*The staff had become quite overwhelmed. We had a lot of staff turnover, a lot of absenteeism. There was a strong belief that the patients had changed in their nature of their level of acuity and the effort that it took to care for them. So we needed…we just knew that we needed some kind of a different model*.

(Unit Leader 1)

#### 3.1.2. Supporting Equitable Workloads

The majority of direct care nurse and leader participants acknowledged concerns with inequitable unmanageable workloads. They provided numerous examples of unbalanced care assignments where some nurses had higher than normal patient care requirements, leaving others with less intense work demands. Participants attributed workload issues to a lack of objective measures for determining nurse assignments based on patient care needs and nurse competencies. One leader stated that without objective data, conflicts often arose among staff, resulting in low morale and high turnover.

*Well, the main reason, in my opinion, was to be able to reflect the workload for the nurse-to-patient ratio-number one. And number two, is to ensure that we have the right nurse caring for the right patient in terms of the skills and competency*.

(Unit Leader 3)

### 3.2. Facilitators to the Implementation of the Synergy Model

The three facilitator themes were: (1) Having a dedicated trusted nurse champion, (2) organizational support, and (3) nurse-driven patient-centered.

#### 3.2.1. Having a Dedicated Trusted Nurse Champion

Across the 12 programs, a component of the implementation was a dedicated champion role over 6 months with responsibility for educating staff about the Synergy Model and conducting audits on reliable synergy patient assessment tool use. Participants identified the importance of having a dedicated nurse champion with protected time throughout the first months of implementation to “smooth” implementation. Participants also described the nurse champion as someone trusted by staff and leadership based on their knowledge of the unit, patients, and staff—someone with “experience”. To reach all nurses and to offer ‘refreshers’ as needed, champion nurses held short in-services, one-to-one sessions, and regular audits of nurses’ synergy scoring.

*We did have a champion…she was an RN at the bedside. And therefore, I think this was helpful because it made the tool, kind of, you know, more realistic, and the feedback that the RN provides is more relevant to the staff so they can make connections as opposed to being enforced by leadership*.

(Unit leader 2)

*I started integrating it [the Synergy Model] as a discussion point…I asked them about their patient assignment. But at the end, I also asked them to include their synergy scores. So, I started reviewing routinely what synergy scoring is and getting them to start to work through identifying the acuity and capability scores for each of their patients*.

(Unit leader 3-acting as a nurse champion)

#### 3.2.2. Organizational Support

This theme was defined as the presence of senior leadership during the roll-out, financial and material support for training, accessible toolkit resources, and ongoing commitment to the initiative beyond the roll-out period.

*So, for staff to be able to know that … one of the chiefs of practice, she was one of the practice chiefs at the time…was attached to it as well as the manager. I think that helps quite a bit for buy-in.*.

(Unit Leader 5)

The organizational roll-out strategy of the Synergy Model included the development of a standardized electronic version of the synergy patient assessment tool—evidence of longer-term investment in the Synergy Model. The electronic standardized version of the assessment tool provided a unified and common language across units using the same eight patient assessment characteristics and the same five-point scale for scoring patients on acuity and capability needs. Considerable pre-work was done by clinical informatics teams, data analytics personnel, nurses, nurse leaders, and educators to develop online resources for Synergy Model implementation prior to roll-out. Thoughtful planning before implementation was key evidence to all participants of the organization’s support for the nursing model of care redesign and long-term sustainability.

*So, she [informatics expert] worked with myself, the unit leader, the educator and our nurse champion to create the corporate tool that would sort of blend the tool we’re using and the corporate tool together and make it meaningful and usable for our team*.

(Direct Care Nurse 2)

#### 3.2.3. Patient-Centered, Nurse-Driven

During the roll-out, senior leadership aligned the use of the Synergy Model with their vision and mission of true patient-centered care. In discussions with direct care nurses and unit leaders, senior leadership described care delivery redesign as a better way to “conceptualize nursing care of patients” with a focus on holistic patient needs and nurse-driven assessment and care planning. Direct care nurses, in particular, appreciated the focus on patients’ acuity and capability needs, and their capacity to use their professional judgment when scoring patients after assessment. Nurses also appreciated the use of the tool to record real-time changes in their patients’ status, and to use synergy scores to plan care of significance to patients and to them.

*…somebody I’m working with changes over the course of the day and becomes unstable, becomes complex, it’s nice to stay with those two patients because you know what you’re doing for the second shift or the third shift with them versus changing it up and having to relearn new people…And so, in times like that, somebody like me who has a bit more experience, is likely to look at that and go, “You know what? They’re all twos [lower acuity] across the board, I’m going to bump that to a three [higher acuity]”*.

(Direct Care Nurse 8)

### 3.3. Barriers to Implementation of the Synergy Model

Participants described barriers to implementation as: (1) Negative perceptions about the model, (2) human resource challenges, and (3) conflicts between standardization and unit-adaptation of the synergy patient assessment tool.

#### 3.3.1. Negative Perceptions of the Model

Nurses’ negative perceptions of the model arose from the organization’s concurrent use of a workload management tool, the Nursing Intensity Management System (NIMS). The NIMS is a tool filled out by nurses at end-of-shift to capture the nursing workload. Some study participants were confused about the purpose for both tools, questioning the “value add” of the Synergy Model. One participant gave an example of how the NIMS captures time needed for specific tasks, such as dressing changes. This participant felt there needed to be more clarity about Synergy Model benefits.

Another transition associated with the Synergy Model was changeover from staffing assignments based on set ratios, such as a 1:5 nurse-to-patient ratio, to staffing assignments determined by real-time patient acuity and capability synergy scores. Charge nurses, in particular, had to learn a new approach to staffing.

A common misperception among nurse participants was that the adoption of the Synergy Model would result in more direct care nurse hiring and greater resource allocation. The Synergy Model approach can highlight where nurse staffing gaps exist, but during roll-out, senior leadership explained how the Synergy Model was meant to optimize the use of available human resources: There were no promises for additional direct care nurses. At HHS, when nurse staffing gaps became more evident with use of the Synergy Model, additional nurses and other resources were not always forthcoming, resulting in nurse frustration and project disengagement.

*And the other piece is that I think that staff also felt with the introduction of Synergy that it would also maybe provide them with more staffing resources. So maybe they thought they may have gotten additional personnel, like additional nurses, especially when the patient scores were higher. And it wasn’t really about that. It was about looking at what is our existing resources and how could we look at using what we have to manage the patient care assignment*.

(Unit Leader 7)

*Like sometimes we try to up-staff, especially, as I said, when I have like transplants and lots of chemo and things like that. But if the site is short, like [name of site], like other units are short a few RNs, they will pull from us no matter what. So, then I don’t…like it doesn’t help us then*.

(Unit Leader 5)

#### 3.3.2. Human Resource Challenges

Despite the benefits of a patient-centered approach to nurse staffing, all the participants raised concerns about chronic nursing shortages and frequent turnover amidst unpredictable patient flow (e.g., admissions, discharges, transfers). Participants felt that the Synergy Model worked best on units with more predictable patient populations, stable direct care nurse composition, and pre-existing clinical care pathways (e.g., perioperative services, surgery).

*If we could do that in a perfect world, then you’ll get manpower with the twitch of a finger, it will work great. I do like in principle what Synergy stands for, but unfortunately in a regional service where you get admissions all kinds of the day, that’s of no help, very much*.

(Unit Leader 1)

#### 3.3.3. Attrition of Nurse Champion

The attrition of nurse champions was a theme associated with human resource challenges, compounded on units with high turnover and many new direct care nurses, placing persistent orientation demands on other nurses and leadership.

*[Name-nurse champion] had had enough! She left, and she was the go-getter of it all. Yeah, oh yeah! It’s kind of dropped off the face of the earth and [Name-new nurse champion] is trying to keep it going*.

(Direct Care Nurse 6)

#### 3.3.4. Confusion between Standardization and Unit Adaptation of the Synergy Model

The organization created an online version of the Synergy Model to standardize the terminology and scoring of patient needs across clinical programs. At the unit level, nurses and their leaders conceived of the Synergy Model as a means to capture individual patients’ priority care needs in real time. Links between organization-level use of synergy score data and unit-level data have been a source of confusion.

*So, we had worked for a few years to develop our Synergy definitions, to have them reflect our population on our site. And when it went to MEDITECH, they became generic again*.

(Unit Leader 4)

### 3.4. Positive Outcomes to Synergy Model Implementation

The three major themes related to positive outcomes were: (1) More equitable patient assignments and improved decision making, (2) improved communication and advocacy for more resources, and (3) improved workplace culture.

#### 3.4.1. More Equitable Patient Assignments and Improved Decision Making

All participants reported that the implementation of the Synergy Model created a transparent process for creating nurses’ assignments that participants described as “more fair”.

*The main positive one is, I think, people understanding why and not being upset, saying, “Well, I got this assignment, you got that assignment. How come?” or people feeling that ng they unfairly had, you know, heavier assignments*.

(Unit Leader 2)

#### 3.4.2. Improved Communication and Advocacy for More Resources

The Synergy Model promoted communication between nurses and leadership and between nurses due to common consistent terminology and scoring of patients’ acuity and capability needs. Participants described how they could use ‘synergy’ language in hand-overs, huddles, and care planning with each other. They also reported how consistent language promoted their capacity to document and to advocate for patients’ needs and for additional nurse resources. Nurse participants felt that synergy language gave them an objective way of describing their workload, rather than using subjective descriptions, such as “too heavy”. Use of synergy language generated a better awareness of patients’ holistic care needs, a major vision and mission goal of organizational leadership.

*When we’re talking as nurses and saying, “Oh, how’s your day going?” “Oh, it’s so heavy today”. You look at the numbers and go, “Wow. Yeah, you’ve got a lot of number threes [moderate acuity] on that piece of paper. A lot of people are really complex”.…It is there in black and white, it’s not a nurse saying, “Oh, I have to walk my patients to the bathroom all the time”*.

(Direct Care Nurse 9)

*We also use the Synergy Model…if we need to up-staff… We could at one point have, you know, four or five, six very, very ill patients on the ward. Then we may need an extra actual RN-level nurse on the ward versus an RPN. And that helps us to give the proof that we need this extra staff at the time*.

(Unit Leader 3)

#### 3.4.3. Improved Workplace Culture

Participants, particularly unit leaders, discussed the contribution of the Synergy Model towards improving workload, teamwork, and overall culture on the unit. Unit leaders noted reductions in the number of direct care nurse complaints about workload and overtime when the unit was able to successfully use synergy scores to acquire more nurses. Leaders also noted a greater understanding among nurses of how the synergy patient assessment tool could contribute to a manageable workload.

*Well, from a positive perspective, we have less staff complaints about workload and greater teamwork, better culture on the floor. I think because of Synergy, they recognize that the synergy tool is being employed to be sensitive to the actual workload of a particular nurse and not just the generic assignment of, you know, “Everybody gets five patients and good luck” kind of thing*.

(Unit Leader 7)

### 3.5. Negative Outcomes to Implementation of the Synergy Model

Although the Synergy Model resulted in several perceived positive outcomes, participants also cited negative outcomes: (1) Increased levels of frustration, (2) increased burden on experienced staff, and (3) change fatigue.

#### 3.5.1. Increased Levels of Frustration

Participants indicated how there were occasions when units were under-staffed with direct care nurses despite synergy patient assessment tool documentation of high acuity patients. Many participants were concerned about the impact of nurse shortages on the safety and quality of patient care, including the possibility of “making mistakes” or “missing essential care”.

*Well, when it works, it works well [laughs]. Then it doesn’t work. Then people are going home frustrated and tired. And then the long-term outcome from that is the young people say I can work somewhere else, less stressful, I’m out of here and all that training time is useless because they’re gone*.

(Direct Care Nurse 8)

#### 3.5.2. Increased Burden on Experienced Staff

Although participants were appreciative of the role of the Synergy Model in creating assignments according to staff competencies and patient care needs, experienced nurses reported feeling burdened by heavy assignments, particularly when the unit included greater numbers of novice staff and/or greater numbers of high acuity patients. They acknowledged how this issue existed pre-Synergy, but they had hoped that use of the Model would help sort out workload burdens for them.

*That being said, as a senior nurse, I sometimes feel like I get crapped on a lot of the time. Because with [XX] years of experience and number [XX] from the top with seniority, I walk in and I just know every shift that I come into work, it is going to suck*.

(Direct Care Nurse 6)

#### 3.5.3. Change Fatigue

Change fatigue was the third negative outcome theme identified by participants. In particular, unit leaders noted that over the course of implementation, other unit-level changes were ongoing, adversely influencing direct care nurses’ compliance with the Synergy Model, resulting in missed or inaccurate patient synergy scores.

*Well, I think because there’s change, you know, people are kind of, you know, what do they call that? Change fatigue. So, again, we chip away every day with compliance and then getting people to score accurately*.

(Unit Leader 4)

## 4. Discussion

The findings of this study support similar research with the Synergy Model: participants valued the nurse-driven patient-centered approach to this care model [5,9,10,11,13,14]. This study further demonstrated how one tertiary care healthcare organization was able to successfully standardize the synergy patient needs assessment tool across 12 different clinical programs while adapting the synergy scoring indicators for specific patient populations, such as cardiology and neurology. Aggregated electronic data within and across programs more accurately informs health human resource planning over different periods of time while real-time data at unit and individual patient levels provides objective patient needs information for collaborative care delivery decision-making among direct care nurses and leadership (MacPhee et al. 2020 [9]).

Although there were barriers to model implementation, such as change fatigue and nurse frustration with chronic nursing shortages, nurses and leadership recognized how the use of the synergy patient assessment tool was able to provide a common patient-centered terminology for identifying and communicating about patient acuity and capability needs. Direct care nurse engagement with the tool resulted in an improved unit culture and raised awareness of leaders’ efforts to advocate for more direct care nurses.

Models of nursing care, such as the Synergy Model, are also known as professional practice models. Professional practice models are an important expression of nurses’ core values, standards of care, and code of ethics: They foreground nurses’ unique body of knowledge and skills, where the patient is the central focus of care provision [19]. Professional practice models are a foundational component of Magnet hospitals, which are internationally recognized as supportive nursing work environments. Comparative studies of Magnet and non-Magnet work environments have shown that Magnet characteristics, such as professional practice models, are associated with increased nursing job satisfaction and organizational commitment, lower burnout rates, and excellent patient care delivery [20,21]. Given global nursing shortages have been exacerbated by COVID-19, organizations and nursing leadership must consider Magnet-like strategies to enhance nurses’ work environments and the quality of nursing care delivery, such as the adoption of professional practice models [22,23]. The Synergy Model has been widely used as a professional practice model within Magnet organizations, given its focus on direct care nurse engagement in patient-centered care delivery, and nurses’ capacity, as self-regulated professionals, to make autonomous decisions about patient care delivery [4,24].

During organizational restructuring at HHS about a decade ago, senior leadership committed to the development and implementation of a professional practice model for nursing. After a comprehensive review of professional practice models in similar practice environments, a decision was made to adopt the Synergy Model. At the time of this decision, the Synergy Model was being successfully implemented in other Canadian contexts [13,14]. This earlier Canadian nursing research with the Synergy Model concluded that implementation of the model resulted in nurse empowerment, given nurses’ control over patient assessment scoring and the use of synergy scores to inform nurse care planning, communications, and staffing plan decisions. Another advantage of Synergy Model implementation in other Canadian acute care settings was the creation of a care delivery redesign toolkit—a blueprint for professional practice model development, implementation, and evaluation. The toolkit has now been published and is open access [25].

Study participants’ primary reasons for adopting the Synergy Model were a growing awareness of the need for a new nursing model of care to address nurse shortages, burnout, and inequitable workloads. HHS leadership used many evidence-informed strategies to ensure successful implementation of the Synergy Model. Facilitation strategies were assignment of trusted dedicated nurse champions; ongoing organizational support for the model, including its integration within the electronic health record; and education and communications promoting the conceptual underpinnings of the Synergy Model. These facilitation strategies are similar to successful professional practice model implementation strategies in the literature [22,24]. Study participants also identified implementation barriers reported in the literature, including negative perceptions of the model; human resource challenges, especially attrition of champions; and confusion between centralized organization-level goals versus unit-level goals, such as adaptation of the model for a specific unit’s patient population [22,24]. Participants acknowledged successes from implementation of the Synergy Model, particularly more equitable workloads, improved communications and advocacy for needed resources, and an improved work culture. These positive outcomes are similar to evaluations of Magnet work environments whose professional practice models foreground patient-centered care and nurses’ valued contributions to patient care delivery [21,23]. The negative outcomes reflect the reality of continuous change, such as frustration, fatigue, and increased burdens of experienced staff who must regularly cope with the ‘churn’ of staff requiring ongoing mentorship and support [26].

Table 2 provides Synergy Model implementation recommendations based on this study’s findings. These recommendations will hopefully result in successful sustainable adoption of the model with minimal negative outcomes, such as change fatigue.

### Limitations

The study setting was one institution covering 12 diverse clinical programs. This qualitative study of 19 informants across the 12 programs may not have captured all aspects of Synergy Model implementation and evaluation. This qualitative component, however, is part of a larger mixed methods study with quantitative survey data that will help triangulate and strengthen this study’s findings.

We did not compare differences between direct care nurses and unit leaders in our analysis of interview data, although we did separate out quotes to differentiate between the two. There were some unique perspectives between the two groups that require further examination.

Due to the small numbers of participants within one organization, our capacity to separate out participants by clinical program and to include demographics was limited by ethics. Hopefully, survey data, which includes demographics, will provide a more granular look at differences in participant responses by nursing characteristics and by clinical program. More research needs to be done to determine the impact of the Synergy Model on cost-effectiveness, service delivery, and patient population outcomes.

## 5. Conclusions

The Synergy Model was implemented in 12 clinical programs at HHS. Senior leadership systematically planned for this new nursing model of care to address ongoing nurse shortages and nursing workload. The Synergy Model was selected as a nursing professional practice model given its focus on patient-centered nurse-driven care, and its use in other Canadian acute care contexts and many US Magnet hospitals. Findings from this qualitative study suggest that implementation of the Synergy Model resulted in more equitable workloads and improvements in nursing culture. Themes from the study also emphasize the importance of ongoing organizational support and investment in nurses’ engagement in decision-making, particularly with respect to assessment and prioritization of patient needs.

## Figures and Tables

**Table 1:** Summary of themes by research question.

**Research Question 1. What are the reasons for adopting the Synergy Model?**
Themes Exploring staffing models Supporting equitable workloads
**Research Question 2. What are the facilitators and barriers during the implementation of the Synergy Model?**
Facilitator Themes: Having a dedicated, trusted nurse champion Organizational support Nurse-driven, patient-centered
Barrier Themes: Negative perceptions of the model Human resource challenges Attrition of champions Confusion over central standardization versus unit adaptation
**Research Question 3. What are the participants’ perceived outcomes after implementation of the Synergy Model?**
Positive Outcomes Themes: More equitable assignments, improved decision making Improved communication and advocacy for more resources Improved workplace culture
Negative Outcomes Themes Increased levels of frustration Increased burden on experienced staff Change fatigue

**Table 2:** Synergy Model implementation recommendations.

**Senior Leadership Recommendations**
− Integrate the synergy patient assessment tool within the electronic health record − Collaborate with leadership and direct care nurses to consider best use of synergy patient assessment data at organizational, unit and individual patient levels − Reinforce the importance of the Synergy Model as a professional practice model that foregrounds nursing contributions to excellent patient care delivery − Provide visible, ongoing commitment to the initiative, including resources for long-term sustainability − Include synergy patient assessment data as a component of accessible visible quality improvement or organizational performance dashboards
**Unit Leaders**
− Collaborate with direct care nurses to use synergy patient assessment data to make real-time staffing decisions − Be transparent and explicit about how synergy patient assessment data are being used to address direct care nurse shortages − Regularly communicate the practical importance of the Synergy Model at staff meetings and huddles
**Nurses**
− Be a champion for enhanced, patient-centered care and the Synergy Model − Participate in identifying and refining synergy patient assessment indicators on the scoring guidelines; participate in periodic reliability checks of nurses’ synergy scores − Advocate for the Synergy Model with colleagues: Explain the importance of having a professional practice model that represents nursing within the organization − Recognize barriers to safe, quality patient care and advocate for innovative change

## Data Availability

Not applicable.

## References

[1] Havens D., Gittell J., Vasey J. (2018). Impact of relational coordination on nurse job satisfaction, work engagement and burnout: Achieving the quadruple aim. J. Nurs. Admin..

[2] Agency for Clinical Innovation (2013). Understanding the Process to Develop a Model of Care: An ACI Framework. https://www.aci.health.nsw.gov.au/__data/assets/pdf_file/0009/181935/HS13-034_Framework-DevelopMoC_D7.pdf#:~:text=%E2%80%9CModel%20of%20Care%E2%80%9D%20broadly%20defines%20the%20way%20health,the%20right%20team%20and%20in%20the%20right%20place2.

[3] Booker C., Turbutt A., Fox R. (2016). Model of care for a changing healthcare system: Are there foundational pillars for design?. Aust. Health Rev..

[4] Curley M.A. (2007). Synergy: The Unique Relationship between Nurses and Patients, the AACN Synergy Model for Patient Care.

[5] Nania T., Barello S., Caruso R., Graffigna G., Stievano A., Pittella F., Dellafiore F. (2021). The state of the evidence about the Synergy Model for patient care. Int. Nurs. Rev..

[6] Carter K.F., Burnette H. (2011). Creating patient-nurse synergy on a medical-surgical unit. Medsurg. Nurs..

[7] Gralton K.S., Brett S.A. (2012). Integrating the Synergy Model for patient care at Children’s Hospital of Wisconsin. J. Pediatr. Nurs..

[8] Ho E., Principi E., Cordon C., Amenudzie Y., Kotwa K., Holt S., MacPhee M. (2017). The Synergy Tool: Making important quality gains within one healthcare organization. Admin. Sci..

[9] MacPhee M., Wagner J., Udod S., Berry L., Perchie G., Conway A. (2020). Using the Synergy Tool to determine Regina Emergency Department staffing needs. Nurs. Leadersh..

[10] Udod S., MacPhee M., Wagner J., Berry L., Perchie G., Conway A. (2021). Nurse perspectives in the Emergency Department: The Synergy Tool in workload management and work engagement. J. Nurs. Manag..

[11] Wagner J., MacPhee M., Udod S., Berry L., Perchie G., Conway C. (2021). Surveys conducted pre-and post-implementation of a Synergy Tool: Giving voice to emergency teams. J. Nurs. Manag..

[12] Khalifehzadeh A., Jahromi M., Yazdannik A. (2012). The impact of the Synergy Model on nurses’ performance and the satisfaction of patients with acute coronary syndrome. Iran. J. Nurs. Midwifery Res..

[13] MacPhee M., Jewell K., Wardrop A., Ahmed A., Mildon B. (2010). British Columbia’s provincial nursing workload project: Evidence to empowerment. Nurs. Leadersh..

[14] MacPhee M., Wardrop A., Campbell C., Wejr P. (2011). The Synergy professional practice model and its patient characteristics tool: A staff empowerment strategy. Nurs. Leadersh..

[15] Sandelowski M. (2000). Whatever happened to qualitative description?. Res. Nurs. Health.

[16] Kim H., Sefcik J.S., Bradway C. (2017). Characteristics of qualitative descriptive studies: A systematic review. Res. Nurs. Health.

[17] Patton M.Q. (2015). Qualitative Research & Evaluation Methods: Integrating Theory and Practice.

[18] Nowell L., Norris J., White D. (2017). Thematic analysis: Striving to meet the trustworthiness criteria. Int. J. Qual..

[19] Glassman K. (2016). Developing and implementing a professional practice model. Nurs. Sci. Q..

[20] Dit Dariel O., Regnaux J. (2015). Do Magnet^®^-accredited hospitals show improvements in nurse and patient outcomes compared to non-Magnet hospitals: A systematic review. JBI Evid. Syn..

[21] Stimpfel A., Rosen M., McHugh M. (2015). Understanding the role of the professional practice environment on quality of care in Magnet^®^ and non-Magnet hospitals. J. Nurs. Admin..

[22] Twigg D., McCullough K. (2014). Nurse retention: A review of strategies to create and enhance positive practice environments in clinical settings. Int. J. Nurs. Stud..

[23] Speroni K., McLaughlin M., Friesen M. (2020). Use of evidence-based practice models and research findings in Magnet-designated hospitals across the United States: National survey results. Worldviews Evid. Based Nurs..

[24] Slatyer S., Coventry L., Twigg D., Davis S. (2016). Professional practice models for nursing: A review of the literature and synthesis of key components. J. Nurs. Manag..

[25] MacPhee M., Havaei F., Fitzgerald B., Budz B., Waller D., Li C., Larmet J. (2020). Care Team Design: Generic. https://open.library.ubc.ca/cIRcle/collections/facultyresearchandpublications/52383/items/1.0389759.

[26] Saarnio R., Suhonen M., Isola A. (2016). Nurse managers’ visions of future challenges in health care organizations. J. Nurs..

